# Cancer incidence in men: a cluster analysis of spatial patterns

**DOI:** 10.1186/1471-2407-8-344

**Published:** 2008-11-25

**Authors:** Tiziana Cassetti, Francesco La Rosa, Luca Rossi, Daniela D'Alò, Fabrizio Stracci

**Affiliations:** 1 Umbrian Population Cancer Registry, Department Medical-surgical Specialties and Public Health, Public Health Section, University of Perugia, Italy

## Abstract

**Background:**

Spatial clustering of different diseases has received much less attention than single disease mapping. Besides chance or artifact, clustering of different cancers in a given area may depend on exposure to a shared risk factor or to multiple correlated factors (e.g. cigarette smoking and obesity in a deprived area). Models developed so far to investigate co-occurrence of diseases are not well-suited for analyzing many cancers simultaneously. In this paper we propose a simple two-step exploratory method for screening clusters of different cancers in a population.

**Methods:**

Cancer incidence data were derived from the regional cancer registry of Umbria, Italy. A cluster analysis was performed on smoothed and non-smoothed standardized incidence ratios (SIRs) of the 13 most frequent cancers in males. The Besag, York and Mollie model (BYM) and Poisson kriging were used to produce smoothed SIRs.

**Results:**

Cluster analysis on non-smoothed SIRs was poorly informative in terms of clustering of different cancers, as only larynx and oral cavity were grouped, and of characteristic patterns of cancer incidence in specific geographical areas. On the other hand BYM and Poisson kriging gave similar results, showing cancers of the oral cavity, larynx, esophagus, stomach and liver formed a main cluster. Lung and urinary bladder cancers clustered together but not with the cancers mentioned above. Both methods, particularly the BYM model, identified distinct geographic clusters of adjacent areas.

**Conclusion:**

As in single disease mapping, non-smoothed SIRs do not provide reliable estimates of cancer risks because of small area variability. The BYM model produces smooth risk surfaces which, when entered into a cluster analysis, identify well-defined geographical clusters of adjacent areas. It probably enhances or amplifies the signal arising from exposure of more areas (statistical units) to shared risk factors that are associated with different cancers. In Umbria the main clusters were characterized by high risks for cancers with alcohol and tobacco both as risk factors. Tobacco-only related cancers formed a separate cluster to the alcohol- and tobacco-related sites. Joint spatial analysis or investigation of hypothesized exposures might be used for further investigation into interesting geographical clusters.

## Background

Umbria is a small region in Central Italy with a population of about 850,000. Well-defined high risk areas exist for some cancer sites (e.g. gastric cancer and upper aero-digestive cancer) in the northern and eastern parts of the region. A descriptive study of cancer incidence and mortality by municipality was conducted using data from the regional population cancer registry (RTUP) and from the regional nominative cause of death registry (ReNCaM) [1,2]. Since cancer data were aggregated at the municipal level, variability due to small areas hampered interpretation of observed SIRs in terms of underlying local cancer risks [3]. Thus the widely used Besag, York, and Mollie spatial analysis method was adopted to produce regional maps by gender and cancer site [4]. These studies provided evidence of marked intra-regional variability in cancer distribution but did not analyze the incidence of diverse cancers simultaneously.

Although recent methods for joint disease mapping were first developed to investigate co-occurrence of two events [5-7], and then extended to more than two events [8], these models are still not well-suited for analyzing many cancers simultaneously. Cluster analysis includes several exploratory techniques that were developed to identify data grouping and to generate hypotheses. It is distinct from spatial analysis methods which investigate "unusual" disease clusters (i.e. events concentrated in time or space that are unlikely to be due to chance alone). In the study of geographical disease distribution cluster analysis is infrequently used, [9] although it is more descriptive than joint spatial modeling, and characterizes local areas where shared factor(s) generate(s) a cluster of cancers. As it is exploratory and quickly identifies latent spatial fields, it may be considered a screening tool for identifying candidate cancer sites that should be included in a joint disease mapping analysis.

In this paper we propose a simple two-step approach that is based on a cluster analysis of municipal SIRs for exploring the pattern of cancer incidence in-depth in sub-regional areas and for establishing correlations among risks of different cancers.

## Methods

Incidence data for the period 1999 to 2003 were obtained from the Umbrian Population Cancer Registry. Population data were provided by the national institute of statistics (ISTAT). In Umbria, 399.162 residents constituted the male population in 2001. Cases were collected, coded, registered and analyzed in accordance with the standard recommended methods for cancer registries [10]. Incidence was coded according to the Tenth International Classification of Diseases (ICDX) [11]. In the Umbrian male population the most common solid cancer sites were the oral cavity and pharynx (C01-C06, C09-C14 ICDX), esophagus (C15 ICDX), stomach (C16), colon-rectum (C18-C21), liver (C22), pancreas (C25), larynx (C32), lung (C33-C34), skin melanoma (C43), prostate (C61), kidney (C63), urinary bladder (C67) and thyroid gland (C73). All bladder cancers were considered malignant if not reported as non-infiltrating.

Standardized incidence ratios by municipality were calculated using the indirect method, with the regional number of cases in the study time-frame as standard [12].

To estimate smoothed SIRs we fitted two different models: the Besag, York e Mollie (1991) [4], which is commonly used in epidemiological studies and which can be implemented using public domain software, and Poisson kriging [13].

### The BYM model

O_i _represents the observed number of cancer cases and E_i _the expected number, calculated using the indirect method in the ith municipality. We assumed that observed cases O_i _are Poisson distributed with the mean depending, through a logarithmic link function, on the expected cases E_i _and on a spatially auto-correlated random effect, that is:

O_i_ ~ *Poisson(μi)*

log(*μ_i_*) = log(*E_i_*) + *β*_0_ + *ϕ_i_*

where μ_i _is the mean of the Poisson distribution, β_0 _is a constant representing the intercept of the (log) relative risk in Umbria, and ϕ_i _is a spatially auto-correlated random effect capturing the residual relative risk in the ith municipality which the intercept does not cover. For the random effects, ϕ_i_, we assumed an intrinsic conditional autoregressive (CAR) model [4]; random spatial effects follow a multivariate normal distribution and the conditional mean of each ϕ_i _is the weighted sum of the other ϕ_i_s. We specified the following 'vague' prior distributions for the other parameters in the model: Gaussian distribution for the intercept parameter β_0 _with mean 0 and precision parameter equal to 1.0E-5; and gamma distribution for the precision parameter of the CAR model with r equal to 1.0E-1 and μ equal to 1.0E-1.

For each cancer site, the BYM was fitted using WinBUGS version 1.41, a standard public domain package for Bayesian inference using Markov Chain Monte Carlo (MCMC) methods.

To assess dependency of clustering on the smoothing technique, we considered the following ATA (area-to-area) Poisson kriging model.

### Poisson kriging model

The risk over a given municipality is a linear combination of the target municipality and the neighboring municipalities.

$${\hat{r}}_{PK}(v_{\alpha })=\sum_{i=1}^{K}\lambda_{i}(v_{\alpha })z(v_{i})$$

where the weights *λ*_*i *_(*v*_*α*_) were calculated according to the formula reported in [13].

We assumed that all municipalities have similar shapes and sizes, with a uniform population density. Each municipality was represented by its centroid *u*_*α *_= (*x*_*α*_, *y*_*α*_).

We also assumed that the number of registered deaths *d*(*v*_*α*_) was a random variable following a Poisson distribution with one parameter given by the population size multiplied by local risk. The Poisson kriging model was fitted using the public domain software "poisson-kriging.exe" described in [13].

Both the non-smoothed SIRs and the SIRs that were smoothed from different models were entered into the cluster analysis. The un-weighted pair group method with arithmetic averages (UPGMA) was adopted. It is one of the most frequently used cluster analysis methods [14,15]. The "r" Bravais-Pearson correlation coefficients was the similarity index. The 92 Umbrian municipalities were first considered as operational taxonomy units (OTUs) and the SIRs of thirteen cancer sites as observations; then the cancer sites were considered as OTUs and the SIRs of municipalities as observations.

## Results

Cluster analysis of non-smoothed SIRs showed only oral cavity and larynx cancers clustered at r = 0.8. No clear clustering emerged among municipalities as the clustering level was very low (highest r = 0.4) and distant areas often clustered together.

**Figure 1:**
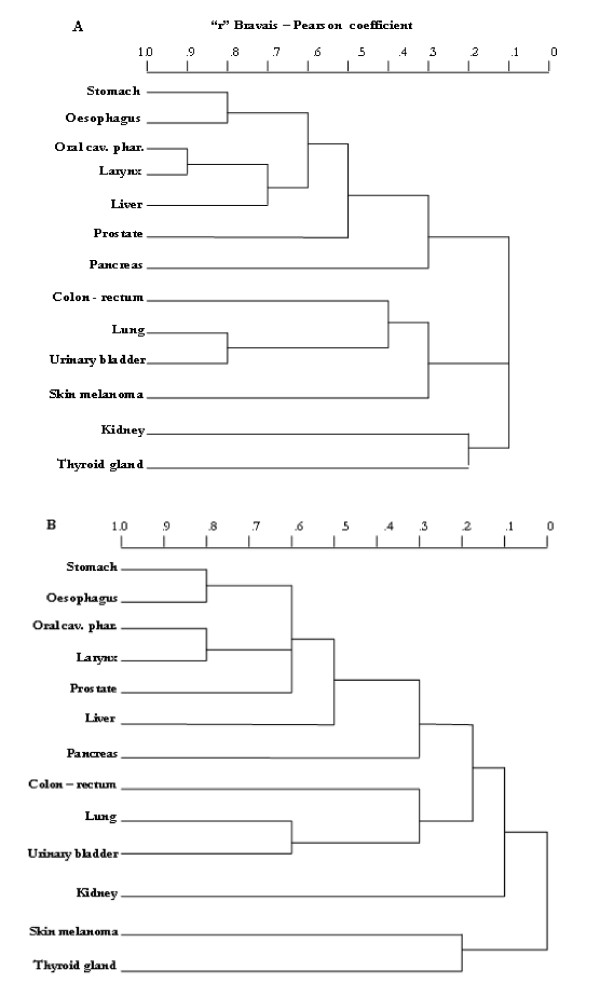
Dendrogram showing the relationships among the thirteen cancer sites based on BYM modeling (upper) and Poisson kriging (bottom) respectively.

The dendrogram in Figure 1 illustrates clustering of the thirteen cancer sites by SIR distribution in the 92 Umbrian municipalities, as obtained from Poisson kriging and BYM modeling respectively.

### BYM derived SIRs

Most marked aggregation involved the sites related to the upper aero-digestive tract and liver. A strong correlation emerged between lung and urinary bladder cancer sites.

**Figure 2:**
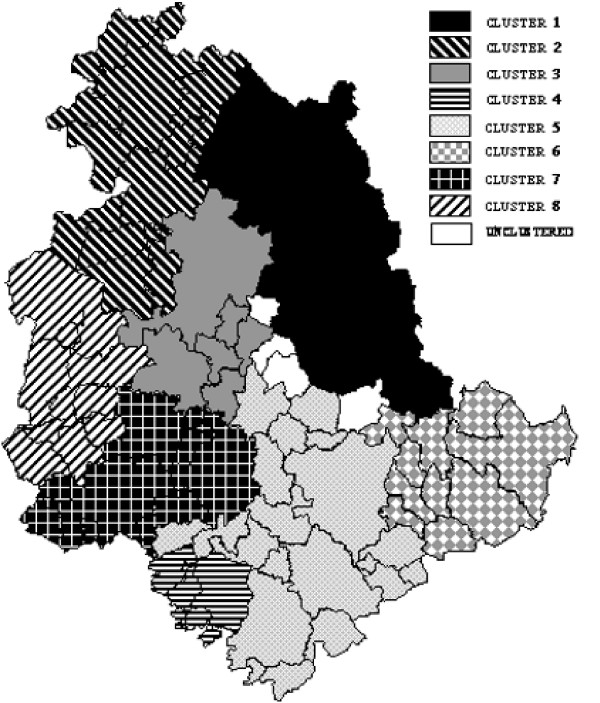
Clustering of the regional municipalities based on risk of different cancers.

Figure 2 shows the geographical distribution of Umbrian municipalities aggregated in eight clusters at the r = 0.5 level, resulting from cluster analysis of BYM smoothed SIRs. Only four municipalities were unclustered, the other 88 clustered in well-defined geographical areas.

Cluster 1: (north-east Umbria), included a high incidence of oral cavity and pharynx, larynx, esophagus and liver cancers and low SIRs for lung, melanoma, urinary bladder and thyroid cancers (table 1).

**Table 1:** Mean smoothed SIRs (BYM model) by cancer sites in eight clusters of regional municipalities.

**Site**	**Cluster 1**	**Cluster 2**	**Cluster 3**	**Cluster 4**	**Cluster 5**	**Cluster 6**	**Cluster 7**	**Cluster 8**
Stomach	99.16	157.50	96.65	89.30	85.35	101.60	91.77	96.11
Esophagus	112.50	155.73	89.20	68.02	79.03	119.05	111.75	103.28
Oral cav. phar.	125.34	121.21	97.79	91.95	80.47	91.04	82.28	95.89
Larynx	114.65	109.74	101.40	103.35	89.90	99.43	89.89	96.71
Liver	106.00	113.16	104.88	104.72	93.98	97.54	96.62	108.91
Prostate	100.26	111.87	101.40	99.33	90.47	87.64	99.82	97.33
Pancreas	102.97	110.38	87.42	104.91	96.83	110.23	91.74	103.23
Colon-rectum	96.69	101.65	93.57	93.26	100.73	106.51	100.61	101.83
Lung	88.46	111.93	102.56	89.23	101.16	99.81	103.40	92.10
Urinary bladder	94.23	108.00	96.15	85.37	100.58	101.05	99.08	91.09
Skin melanoma	85.57	106.39	99.92	89.89	102.37	88.98	92.82	106.65
Kidney	104.42	98.95	98.16	92.26	102.60	109.22	102.74	98.29
Thyroid gland	94.30	94.25	106.20	111.20	106.99	100.53	116.37	102.42

Modeling (upper) and Poisson kriging (bottom) respectively.

Cluster 2: (north-west): all sites presented a SIR over 100, excluding kidney (98.95) and thyroid (94.25). Cluster 3, which includes Perugia, regional capital and largest town in Umbria, showed the majority of SIRs fell between 95 and 105. Only thyroid cancer was over 100 while esophagus, pancreas and colorectal cancer were lower.

Cluster 4: (south-west with seven villages), showed SIR values were distributed in a reverse pattern to the north-east cluster. Only the thyroid cancer SIR was quite high (111.20).

Cluster 5: (south-central) included the town of Terni and nineteen other municipalities. The upper aero-digestive tract and prostate cancers presented low SIR values.

Cluster 6: (eastern mountainous zone with 10 small villages) presented low SIR values for prostate, melanoma, oral cavity and pharyngeal cancers. Values were high for esophagus, pancreas and kidney cancers.

Cluster 7: (south-west) presented a high SIR for thyroid cancer and low SIRs for oral cavity and pharynx, stomach, larynx and pancreas cancers and skin melanoma.

Cluster 8 (west, around Lake Trasimeno) showed high SIRs for liver cancer and skin melanoma and low values for lung and urinary bladder cancers.

Clustering of municipalities was similar in the Poisson kriging and BYM models. In the Poisson kriging model the north-western, north-eastern and south-eastern clusters clearly emerged as in the BYM model but the clustering level was lower and clusters frequently contain non-neighbouring municipalities.

### Poisson kriging

Similar clustering by cancer site was observed in the geostatistical model with weaker correlations.

**Figure 3:**
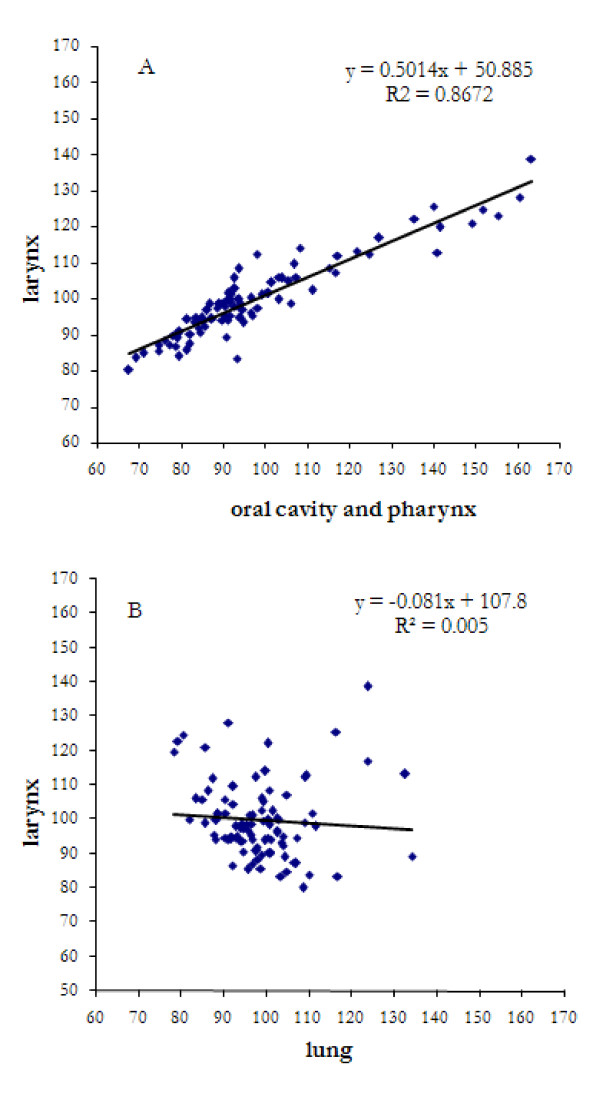
Correlation between smoothed larynx cancer SIRs (BYM model) and those of the oral cavity and pharynx (upper), and lung (bottom) respectively.

Figure 3 shows a significant correlation between the SIRs for larynx and oral cavity cancers and pharynx cancers but not between larynx and lung cancers.

## Discussion

Joint analysis of cancer incidence is mainly concerned with generating and corroborating hypotheses on exposures [8]. The fingerprint of a given exposure may be clustering of cancers sharing a common risk factor. Clustering may also depend on exposure to a proximal factor such as socioeconomic status associated with risk factor distribution for different cancers, chance or artifact.

In this paper, we propose a two-step method (SIR calculation followed by cluster analysis) for exploring cancer site clusters and for characterizing risk patterns in sub-regional areas. To ascertain the best method for cluster detection we compared non-smoothed SIRs, BYM, and Poisson kriging smoothed SIRs.

Cluster analysis of non-smoothed SIRs was almost non-informative because only closely correlated cancer sites, e.g. larynx and oral cavity, clustered together. No geographical clusters emerged from the analysis of municipalities probably because of small area variability, which causes misleading mapping even when a single cancer is considered [16], and reduces correlations among cancer sites. The effect of small area variability is much more marked when similarities are sought concomitantly in the incidence patterns of many cancers rather than when a single disease is of interest.

The much more informative BYM and geostatistical models yields similar results. BYM smoothing produced more homogeneous geographical areas than Poisson kriging, confirming it yielded smoother risk surfaces [17]. Since the SIRs for each cancer site were modeled using vague priors, and independently of other sites, identified clusters seem unlikely to be artifacts consequent to modeling assumptions. As BYM appears to enhance or even amplify the signal from composite areas with increased/decreased risk for a given cancer, it seems best suited for investigating patterns of co-occurrence of different cancers. On the other hand, Poisson kriging may be more suitable for identifying localized single disease clusters i.e. a single area with unusual rates that are hidden in the BYM model (false negatives).

If we look at the BYM results of geographical clustering, the most interesting findings emerged from Clusters 1, 2 and 8. Municipalities in cluster 2 (north-west Umbria) stand out for clusters of stomach and esophagus cancers, followed by oral cavity and pharynx, liver, prostate, pancreas and lung cancers. Only kidney and thyroid cancers showed SIRs which were just below 100 in this cluster. At the beginning of the 1980s, a very high incidence of gastric cancer which was mainly related to dietary factors [18,19], and approached Japanese rates [20], was observed in this area of Umbria. In fact, it is part of a known high risk area in central Italy that includes the provinces of Forlì in the Romagna region, Arezzo in Tuscany and Pesaro in the Marches. Umbrian municipalities in cluster 1 also had high rates of cancer sites linked to joint consumption of alcohol and tobacco. A recent survey of the four local health districts in the Umbria Region reported a significantly higher prevalence of binge drinkers in district n.1, which includes municipalities in clusters 1–2 [21]. SIRs for gastric cancer, although high, were lower than in cluster 2.

The high incidence of skin melanoma in the municipalities around Lake Trasimeno (cluster 8) could be related to intermittent sun exposure during the summer rather than to widespread opportunistic screening.

In the present analysis, the main cluster of cancer sites included the oral cavity and pharynx, larynx, esophagus, liver, and stomach (r = 0.6), most of which are related to the synergistic effect of alcohol and tobacco consumption [22]. Although alcohol consumption alone was not significantly associated with the risk of gastric cancer [23], present results are divergent as they show a strong association of gastric cancer with alcohol-related sites, particularly with the esophagus (r = 0.8). Clustering of gastric and esophageal cancer may also reflect an association between esophagus adenocarcinoma and gastric cardia cancer, which was reported to be independent of *Helicobacter pylori *infection [18,24]. Cancer of the liver was less strongly correlated and, in fact, tobacco and alcohol were reported to act as independent risk factors in liver cancer [25].

Another finding which emerged from the present analysis was a weak (r = 0.5) association of alcohol-related sites with prostate cancer. As the incidence of prostate cancer is largely influenced by local use of opportunistic screening [26], co-occurrence of high rates of prostate cancer and alcohol-related sites without shared risk factors may be hypothesized. In fact, in a case-control study Chang et al. detected no association between recent alcohol consumption and risk of advanced, sporadic, or familial prostate cancer, but found a positive borderline association with localized disease [27].

Lung cancer, the most important tobacco-related site, clustered with urinary bladder cancer but was very distant (r = 0.1) from the main cluster. The occurrence of larynx cancer was strictly related to head and neck, but not with lung cancer (figure 3). Moreover, some municipalities' clusters were characterized by high SIRs of larynx cancer and low SIRs of lung cancer, and vice versa. A low spatial correlation between lung cancer and tobacco- and alcohol-related cancer sites was reported by Knorr-Held et al. using joint spatial analysis [8].

## Conclusion

In conclusion, results in terms of exposures should be interpreted with caution as the hypotheses this study generated require further confirmation. Furthermore, one limitation of our study may lie in our choice of average linkage clustering from among the many cluster analysis techniques that are currently available. The UPGMA method, although one of the most widely used, depends on the initial cut, i.e. on the selection of the first cluster, and may sometimes yield suboptimal clustering results for a given dataset [28]. Ongoing research is assessing the roles of the clustering method and CAR modeling assumptions (e.g. assuming different priors) in determining geographical cluster formation.

Despite this, results are in good agreement with local data on risk factors and other reports. The present application to cancer incidence in an Italian region produced evidence of separate clustering among alcohol- and tobacco-related cancers and yielded interesting patterns of cancer incidence. Our simple two-step method for screening clusters of different cancer sites may prove to be a useful addition to single disease mapping and joint spatial analysis, to be used when grouping many diseases.

## Competing interests

The authors declare that they have no competing interests.

## Authors' contributions

TC drafted the manuscript. FLR conceived the idea and study design and contributed to statistical analyses. DD acquired and revised data. LR carried out statistical analyses. FS interpreted data and critically revised the manuscript.

## Pre-publication history

The pre-publication history for this paper can be accessed here:


